# rs2572431 Polymorphism on Chromosome 8 Is Associated With Individual Differences in Anxiety Related Coping Modes

**DOI:** 10.3389/fpsyg.2019.01451

**Published:** 2019-07-09

**Authors:** Sonja Jung, Cornelia Sindermann, Bernd Lachmann, Christian Montag

**Affiliations:** Department of Molecular Psychology, Institute of Psychology and Education, Ulm University, Ulm, Germany

**Keywords:** rs2572431, coping, vigilance, cognitive avoidance, neuroticism

## Abstract

**Objective:**

The role of genetic factors in the interplay between anxiety-related coping and personality has been the subject of interest in numerous previous studies. The current study focused on anxiety-related coping modes, namely repression versus sensitization (i.e., cognitive avoidance versus vigilance), and the single nucleotide polymorphism (SNP) rs2572431. An association between this SNP and anxiety-related personality traits has previously been shown in a genome wide association study, thus further investigation of the relationship between this SNP and anxiety-related coping seems warranted.

**Methods:**

In the present study, *N* = 880 mostly Caucasian participants (*n* = 269 males and *n* = 611 females; mean-age: 23.88, *SD* = 7.19) filled in a personality questionnaire assessing individual differences in cognitive avoidance and vigilance, and all participants were genotyped for rs2572431.

**Results:**

Participants homozygous for the T-allele in rs2572431 showed the highest vigilance scores in all scenarios tested. This is in line with findings from an earlier genome wide association study demonstrating that the T-allele is also associated with higher neuroticism scores.

**Conclusion:**

The current study yields evidence for the role of rs2572431 in the molecular genetic underpinnings of coping modes and, more broadly, for its connection with personality.

## Introduction

The heritability of coping styles has been previously investigated in numerous twin studies (e.g., Kendler et al., 1991; Mellins et al., 1996; Busjahn et al., 1999; Kato and Pedersen, 2005; Kozak et al., 2005; Jang et al., 2007). These studies demonstrated the importance of both genetic and environmental influences for better understanding individual differences in coping styles. For example, Kato and Pedersen (2005) found a moderate additive genetic influence (estimates of percentage of total variation: 0.15 for men; 0.48 for women) for the coping style *Avoidance*, as measured by the Stress Coping Questionnaire (Billings and Moos, 1984). For females only they also detected shared environmental influence (estimate of percentage of total variation: 0.14) for this coping style. In their review, Dunn and Conley (2015) reported the highest heritability estimates (0.68–0.76.) for non-additive genetic factors in the context of coping, underlining the importance of genetic interactions in the heritability of coping styles.

In the context of molecular psychology, a number of candidate gene association studies aiming to illuminate individual differences in personality have investigated polymorphisms related to the serotonergic (Cicchetti et al., 2007; Wilhelm et al., 2007; Szily et al., 2008), dopaminergic (Cicchetti et al., 2007) and adrenergic (Busjahn et al., 2002; Poole et al., 2006) system. More specifically, polymorphisms from the serotonergic system, such as 5-HTTLPR (which stands for serotonin transporter-linked polymorphic region), or the dopaminergic system, such as COMT Val158Met^1^, are well-known candidates in genetic association studies investigating the molecular genetic underpinnings of personality (e.g., Flory et al., 1999; Eley et al., 2003; Gonda et al., 2009; Montag et al., 2011, 2012; Montag and Reuter, 2014; Sindermann et al., 2018) and/or psychological health (e.g., Caspi et al., 2003; Jacob et al., 2005; Pezawas et al., 2005; Liberzon et al., 2014). Some of these studies have also underlined the complex interplay between genetic variables and stress coping, helping to understand an individual’s vulnerability to psychiatric disorders (see also Karg et al., 2011 for a meta-analysis on the interaction between 5-HTTLPR and stress in relation to the development of depression).

The main idea behind these association studies could be summed up with the term “candidate gene approach”. In this approach, it is assumed that individual differences in regions of the genetic code that play a role in the manifold processes linked to neurotransmitter metabolism might also have an influence on personality and human behavior. For example, as selective serotonin reuptake inhibitors (SSRIs) improve depressive symptoms, genetic variations of the gene coding for the serotonin transporter (called SLC6A4), where SSRIs bind, might also play a role in understanding individual differences in personality traits linked to negative emotionality (having higher neuroticism is linked with a higher likelihood of also suffering from depression, Lahey, 2009).

In relation to the aforementioned examples of 5-HTTLPR and COMT Val158Met, firstly, 5-HTTLPR describes a genetic variation on the SLC6A4 gene known to result in lower or higher serotonin uptake (e.g., Heils et al., 1996; Hu et al., 2006; Goldman et al., 2010). This genetic variation indirectly impacts on serotonin levels in the synaptic cleft due to the different density of the serotonin transporters at the presynapse, and as a consequence might play a role in the development of personality traits such as neuroticism (see, e.g., Gonda et al., 2009). Secondly, the exchange of only one base results in the role of the polymorphism COMT Val158Met^1^ in different activities of the catechol-*O*-methyltransferase (COMT), and therefore in lower or higher catabolization of dopamine (Boudikova et al., 1990; Lachman et al., 1996; Domschke et al., 2004). Given the paucity of dopamine transporters in the prefrontal cortex (PFC), the COMT Val158Met polymorphism is prominent in having a particularly strong impact on dopaminergic neurotransmission in the PFC (for a COMT review, see Montag et al., 2012).

Much of the published work in the field of personality neuroscience has studied the Big Five of Personality/Five Factor Model of Personality (e.g., McCrae and Costa, 1997), or individual differences in temperament and character according to Cloninger’s psychobiological model (Cloninger et al., 1993). Beyond this, much fewer studies have examined the molecular genetic underpinnings of primary emotional systems impacting on the Big Five of Personality in a bottom-up fashion (for an overview and further detail on this, see Montag and Panksepp, 2017; Montag and Davis, 2018).

To the best of our knowledge, no published study has yet investigated a personality construct relevant to mental health grounded in the classic psychoanalytical tradition – namely, repressor versus sensitizer in the context of molecular genetics. The concepts of repression and sensitization are theoretically based on the psychoanalytic approach of defense mechanisms first described by Freud (1894), and further developed by his daughter Freud (1936). The aim of this ubiquitous and automated (Hoffmann and Hochapfel, 2009) mechanism is the reduction of anxiety (Freud, 1936; Krohne, 1996). Anxiety can be triggered both by challenging external events and disturbing internal psychological processes (Freud, 1936). In this context, repression [or blunting (Miller, 1987), rejection (e.g., Mullen and Suls, 1982), cognitive avoidance (e.g., Egloff and Krohne, 1998)] describes the removal of attention from the anxiety-provoking stimulus, whereas sensitization [or monitoring (Miller, 1987), attention (Mullen and Suls, 1982), vigilance (Egloff and Krohne, 1998)] describes an enhanced perception and processing of threatening information. Similarities and differences between the theoretical concepts of defense mechanisms and coping styles have been previously discussed (e.g., Kramer, 2010), but are still not well understood so far. Typically, defense mechanisms are seen as a special form of coping used when the individual is confronted with anxiety linked stressors (e.g., Krohne, 1996). In contrast to Freud’s state-oriented approach to defense mechanisms, the concepts repression vs. sensitization/cognitive avoidance vs. vigilance are usually seen as trait-orientated personality constructs (Krohne, 2001). The Model of Coping Modes (e.g., Krohne, 1996; Egloff and Krohne, 1998) suggests the link between coping modes and personality can be established by a habitual tendency to use either mainly vigilant or cognitive avoidant strategies when the individual is confronted with threat (Krohne, 2001). It is postulated that this tendency is grounded in a person’s susceptibility to uncertainty (vigilance) or emotional arousal (cognitive avoidance) (Krohne, 2001). In line with this assumption, the link between vigilance and cognitive avoidance, and classic personality constructs such as the Big Five (e.g., McCrae and Costa, 1997), has also been previously described. Amongst others, Egloff and Krohne (1998) found positive correlations between neuroticism and vigilance across all vigilance subscales, and negative associations between neuroticism and cognitive avoidance, again across all cognitive avoidance subscales (this is consistent with our observations in the current study; please see Supplementary Material). Significant positive correlations for extraversion were only found with cognitive avoidance, but not with vigilance. Significant positive associations with conscientiousness were also found for cognitive avoidance, but not for all subscales.

The current study sought to investigate the molecular genetic basis of individual differences in the constructs of cognitive avoidance and vigilance (operationalized as traits). Despite operationalizing these constructs as traits in this study, we acknowledge that the potential fluctuation of defense mechanisms/coping behavior over time has long been a matter for discussion (see, e.g., Labouvie-Vief et al., 1987; Blanchard-Fields and Irion, 1988). In particular, changes in defense mechanisms across age or via therapeutic interventions have been previously discussed (Cramer, 2007; Perry and Bond, 2012). These studies question the trait-orientated approach to defense mechanisms, and indeed fit better with Freud’s (1894) state-orientated point of view. We believe that the investigation of the molecular genetic basis of these coping mechanisms can potentially help to shed more light on the nature of coping strategies, including their associations with personality traits.

In the current study, we focused on a single nucleotide polymorphism (SNP) called rs2572431 as a new possible genetic candidate for coping modes. This SNP lies on chromosome 8 (8p23.1; Luciano et al., 2018). Genetic variations of chromosome 8, going beyond the aforementioned SNP, have previously been linked with neuropsychiatric disorders (e.g., Middeldorp et al., 2009; Tabares-Seisdedos and Rubenstein, 2009), and with neuroticism (e.g., Smith et al., 2016). This SNP has been chosen for the current study because Okbay et al. (2016) detected an association between the T-allele in rs2572431 and higher order neuroticism scores (i.e., negative emotionality) in a genome wide association study. This finding was subsequently replicated by Luciano et al. (2018) in a further large-scale study, which leads to our hypothesis that this SNP could also be associated with the constructs of cognitive avoidance vs. vigilance due to its link with neuroticism (for further explanation see above). Egloff and Krohne (1998) found correlations between 0.29 and 0.33 for the different subscales of vigilance and neuroticism, indicating shared variance between 8 and 11%. For the different subscales of cognitive avoidance and neuroticism, they found correlations ranging between −0.37 and −0.50, indicating shared variance between 14 and 25%. Based on this research, we expected an association between the effect-allele (T) in rs2572431 and cognitive avoidance and vigilance [coping modes, according to Krohne (1993)], the latter of which have already been linked with neuroticism, anxiety and negative emotionality (Egloff and Krohne, 1998). In sum, we expect the T-allele to be linked with higher vigilance scores and lower cognitive avoidance scores.

## Materials and Methods

### Participants

*N* = 893 participants (*n* = 273 males and *n* = 620 females; mean-age: 23.86, *SD* = 7.15, mostly Caucasian) gave electronic and written informed consent to participate, completed the ABI self-report questionnaires (see Questionnaires section) online, and provided buccal swabs for genotyping the rs2572431 polymorphism. Most of the participants were university students. All participants were part of the Ulm Gene Brain Behavior Project (UGBBP) and were recruited through lectures and seminars, notice boards and flyers at Ulm University, and via online advertising through the website of the institute. The UGBBP is a large-scale research project focusing on the genetic background of personality and emotionality. Beside the questionnaire and gene polymorphism mentioned above, additional questionnaires and genetic data were collected. The study was approved by the local ethics committee at Ulm University, Ulm, Germany. Three participants had to be excluded due to missing questionnaire data, and another ten participants were excluded due to missing genotyping data. The final data set consisted of 880 participants (*n* = 269 males and *n* = 611 females; mean-age: 23.88, *SD* = 7.19).

### Material

#### Questionnaires

To assess the vigilance and cognitive avoidance coping modes, we used a stimulus-response inventory, the *Angstbewältigungs-Inventar* (ABI, Egloff and Krohne, 1998). The ABI represents the German version of the Mainz Coping Inventory (MCI, Krohne et al., 2000). The ABI is based on the Model of Coping Modes (Krohne, 1993), and includes two subscales. The subscale ABI-E consists of four fictitious situations representing ego-threat (a speech, exam, job interview and mistake on the job), and the subscale ABI-P consists of four situations representing physical threat (a dentist visit, being approached by a group of strangers in the evening, driving with an inexperienced driver, a turbulent flight). The participants have to read through each scenario (e.g., “*Imagine that you are walking alone through town in the late evening. A group of people, who look suspicious, approach you from out of a side street*” Krohne et al., 2000, p. 299), and imagine being in this situation themselves.

For each of these fictitious scenarios, participants needed to rate 10 different strategies on a dichotomous scale (1 = “applicable”, or 0 = “not applicable”). Five vigilant (e.g., “*I monitor the people precisely*”) and five cognitive avoidant coping strategies (e.g., “*I say to myself, that these people are probably totally harmless*”) were presented for each scenario, allowing separate assessment of the different coping modes: vigilance in the ego-threat scenarios (VIG-E) and the physical threat scenarios (VIG-P), and cognitive avoidance in the ego-threat scenarios (CAV-E) and in the physical threat scenarios (CAV-P) (Egloff and Krohne, 1998; Krohne et al., 2000). Answers were summed separately for every subscale and total scores for vigilance and cognitive avoidance were calculated via a total sum score for vigilance (VIG-T), and a total sum score for cognitive avoidance (CAV-T). Acceptable internal consistencies were found for all subscales and the total scores, with Cronbach’s alphas ranging between 0.73 and 0.86 in this sample.

#### Genotyping

Buccal cells were collected via cotton swabs that were rubbed at oral mucosa and then washed out in an alcoholic solution. This solution was concentrated afterward, e.g., by means of centrifugation, and later used for DNA extraction. Genomic DNA was extracted automatically from buccal cells using the MagNA Pure 96 System and a commercial extraction kit (MagNA Pure 96 DNA Kit; Roche Diagnostics, Mannheim, Germany). Genotyping of the SNP rs2572431 was performed by a collaborating company (Varionostic, Ulm, Germany) using MassARRAY^®^ technology (Agena Bioscience, Hamburg, Germany).

### Procedure

An e-mail address was provided on the recruiting flyers, posters and on our website for individuals that were interested in participating in the study. After giving their permission to participate, a date for a personal appointment was arranged. The personal appointment was necessary so participants could provide their written informed consent and buccal swabs, and to pay the participants for their involvement in the study. In addition, a link to online questionnaires was sent to the participants. Participants were told to complete the online questionnaires before buccal swab collection to ensure complete data sets. This was checked before collection of the buccal swabs.

The online study was programmed with help of the SurveyCoder tool (ckannen.com). After collecting the buccal swabs, samples were concentrated, centrifuged and cooled. The cells were prepared for the extraction of DNA in batches of 95 samples. An aliquot of the solution was pipetted into a 96-well MagNA plate with one negative control. DNA was then automatically extracted and purificated. DNA plates were sent to our collaborating partner for SNP analysis. Questionnaire data and genetic data were matched and used for later analyses.

### Statistical Analyses

The ABI variables were found to be normally distributed after visual inspection of the histograms and consideration of kurtosis and skewness (Miles and Shevlin, 2001). We used Analysis of Variance (ANOVA) to examine gender effects on the ABI variables, and we calculated Pearson correlation coefficients to find effects of age on the ABI variables. Analysis of Variance was conducted with the ABI variables as dependent variables and the SNP information (rs2572431) as the independent variable. The analyses were carried out on genotype level (hence testing the TT vs. CT vs. CC groups). Given that gender influences the ABI variables (e.g., Egloff and Krohne, 1998), we also examined gene by gender effects, controlling for age effects when necessary. Based on our hypothesis the *p*-values from the tests on main effects of genotype on the ABI variables were derived from one-sided testing. All other *p*-values are derived from two-sided testing.

## Results

### Hardy-Weinberg-Equilibrium

The genotype distribution was as follows: CC = 144, CT = 426, and TT = 310. The distribution was in Hardy-Weinberg-Equilibrium (χ^2^ = 0.01, *p* = 0.908).

### Age, Gender, and ABI Variables

Neither the ABI-VIG variables (VIG-E: *r* = −0.001, *p* = 0.968; VIG-P: *r* = −0.02, *p* = 0.636; VIG-T: *r* = −0.01, *p* = 0.771), nor the ABI-CAV variables (CAV-E: *r* = 0.05, *p* = 0.144; CAV-P: *r* = 0.03, *p* = 0.380; CAV-T: *r* = 0.05, *p* = 0.162) were significantly associated with age. Therefore, age was not taken into account in later analyses.

As expected, ANOVA revealed significant effects of gender on all ABI-VIG variables [VIG-E: *F*(1,878) = 19.95, *p* < 0.001; VIG-P: *F*(1,878) = 32.85, *p* < 0.001; VIG-T: *F*(1,878) = 33.68, *p* < 0.001], and all ABI-CAV variables [CAV-E: *F*(1,878) = 22.33, *p* < 0.001; CAV-P: *F*(1,878) = 10.16, *p* = 0.001; CAV-T: *F*(1,878) = 22.38, *p* < 0.001], with females scoring higher than males on all VIG scales, and lower than males on all CAV scales. Thus, given the possibility of gene-by-gender interactions, gender was included as a second independent variable in all calculated models. Descriptive statistics on the vigilance and cognitive avoidance values are shown in Table 1.

**TABLE 1:** Mean scores (standard deviations) of all ABI (sub-)scales, for the total sample and for the male and female subsample.

	**Total (*N* = 880)**	**Male (*n* = 269)**	**Female (*n* = 611)**
Vigilance (ego-threat)	13.19 (4.03)	12.28 (4.35)	13.59 (3.82)
Vigilance (physical threat)	10.45 (4.05)	9.29 (3.97)	10.96 (3.99)
Vigilance (total score)	23.63 (7.13)	21.57 (7.45)	24.54 (6.79)
Cognitive avoidance (ego-threat)	10.32 (3.98)	11.27 (4.12)	9.91 (3.84)
Cognitive avoidance (physical threat)	11.95 (3.60)	12.53 (3.53)	11.70 (3.61)
Cognitive avoidance (total score)	22.28 (6.42)	23.80 (6.60)	21.60 (6.22)

### Vigilance and rs2572431

**FIGURE 1:**
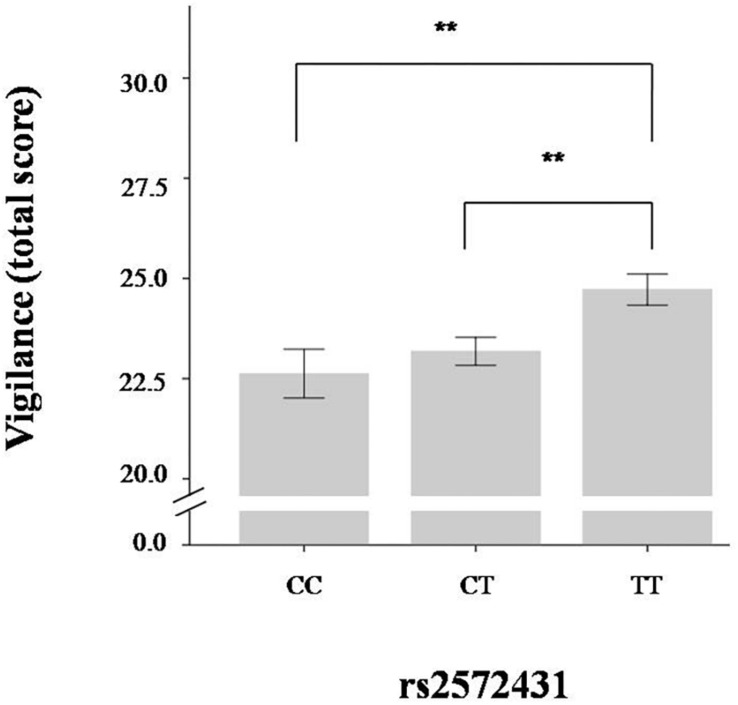
Total vigilance scores according to rs2572431 genotype (^∗∗^*p* < 0.01, derived from *post-hoc* test; error bars indicate standard error).

The multifactorial (gender and genotype as independent variables) ANOVAs revealed significant differences among the three genotype groups for vigilance in the ego-threat scenarios [*F*(2,874) = 5.21, *p* = 0.003, *η*^2^ = 0.01], the physical threat scenarios [*F*(2,874) = 4.72, *p* = 0.005, *η*^2^ = 0.01], and the vigilance total score [*F*(2,874) = 6.45, *p* < 0.001, *η*^2^ = 0.01]. Homozygote carriers of the effect allele (TT group) scored higher on vigilance than those carrying one or two C-alleles (see Table 2). We used G^*^Power v3 to calculate the statistical power of these results. A small *η*^2^ of 0.01 was used in this analysis. A sufficient level of power to detect small effect sizes of 0.76 was calculated *post hoc* for our sample. Although we had a directional hypothesis and therefore did not need to correct our findings for multiple testing, we nonetheless checked how this would affect the results of these analyses: A Bonferroni adjustment would result in an alpha of 0.008 (alpha of 0.05 divided by three tests for avoidance, and three tests for vigilance scenarios). All effects observed with respect to rs2572431 and the vigilance scores are below this threshold. The results of the vigilance total scores are presented in Table 2 and Figure 1.

**TABLE 2:** Mean scores (standard deviations) for vigilance (sub-)scales by genotype for SNP rs2572431.

	**rs2572431 (*N* = 880)**		
	
	CC (*n* = 144)	CT (*n* = 426)	TT (*n* = 310)
Vigilance (ego-threat)	12.65 (4.11)	12.93 (4.10)	13.79 (3.83)
Vigilance (physical threat)	9.97 (3.96)	10.25 (4.11)	10.93 (3.98)
Vigilance (total score)	22.63 (7.29)	23.18 (7.19)	24.72 (6.85)

As mentioned above, main effects of gender were detected for the vigilance subscales [VIG-E: *F*(1,874) = 19.83, *p* < 0.001, *η*^2^ = 0.02; VIG-P: *F*(1,874) = 30.30, *p* < 0.001, *η*^2^ = 0.03] and the total score [VIG-T: *F*(1,874) = 32.20, *p* < 0.001, *η*^2^ = 0.04]. Females showed significantly higher vigilance scores than males across all three vigilance measures. Gender was therefore taken into account as a second independent variable to search for gene-by-gender effects. These were not found for any of the vigilance subscales [VIG-E: *F*(2,874) = 0.65, *p* = 0.522, *η*^2^ = 0.00; VIG-P: *F*(2,874) = 1.45, *p* = 0.236, *η*^2^ = 0.00], or for the total score [VIG-T: *F*(2,874) = 1.09, *p* = 0.337, *η*^2^ = 0.00].

### Cognitive Avoidance and rs2572431

We did not observe a significant influence of rs2572431 on cognitive avoidance for the ego-threat scenarios [*F*(2,874) = 1.08, *p* = 0.170, *η*^2^ = 0.00], for the physical threat scenarios [*F*(2,874) = 0.37, *p* = 0.346, *η*^2^ = 0.00], or for the cognitive avoidance total score [*F*(2,874) = 0.76, *p* = 0.233, *η*^2^ = 0.00]. However, CC-carriers descriptively showed the highest cognitive avoidance scores across all conditions (see Table 3).

**TABLE 3:** Mean scores (standard deviations) for cognitive avoidance (sub-)scales by genotype for SNP rs2572431.

	**rs2572431 (*N* = 880)**		
	
	CC (*n* = 144)	CT (*n* = 426)	TT (*n* = 310)
Cognitive avoidance (ego-threat)	10.57 (3.83)	10.36 (4.13)	10.15 (3.84)
Cognitive avoidance (physical threat)	12.13 (3.64)	11.84 (3.62)	12.02 (3.57)
Cognitive avoidance (total score)	22.70 (6.58)	22.21 (6.54)	22.17 (6.19)

Again, main effects of gender were found for all cognitive avoidance subscales [CAV-E: *F*(1,874) = 20.22, *p* < 0.001, *η*^2^ = 0.02; CAV-P: *F*(1,874) = 8.75, *p* = 0.003, *η*^2^ = 0.01], and the total score [CAV-T: *F*(1,874) = 19.89, *p* < 0.001, *η*^2^ = 0.02]. Females showed lower cognitive avoidance scores than males across all conditions.

No gene-by-gender interactions were found for either the cognitive avoidance subscales [CAV-E: *F*(2,874) = 0.98, *p* = 0.376, *η*^2^ = 0.00; CAV-P: *F*(2,874) = 0.63, *p* = 0.534, *η*^2^ = 0.00], or for the total score [CAV-T: *F*(2,874) = 1.10, *p* = 0.333, *η*^2^ = 0.00].

## Discussion

As hypothesized, given the existing GWAS-findings linking the T-allele of the rs2572431 SNP to neuroticism (Okbay et al., 2016), in the current study we found an association between the rs2572431 risk allele and the neuroticism-related construct of vigilance. More specifically, we found significantly higher vigilance scores for homozygous T-allele carriers in both threat scenarios and for the total scores. As positive links between the T-allele in rs2572431 and neuroticism (Okbay et al., 2016; Luciano et al., 2018), and between vigilance and neuroticism (Egloff and Krohne, 1998), have been observed previously, our results suggest that the rs2572431 polymorphism impacts on the shared variance of the constructs of neuroticism and vigilance. Unfortunately, we did not use the same neuroticism scale in the present study that has been used in the studies highlighted above. Moreover, we did not assess neuroticism with the same questionnaire across all participants in the present data bank (UGBBP) due to conceptual changes (concerning other projects in our department) during the data collection for the current study. Therefore, we can only present analyses of a subsample (*n* = 638) that provided data on rs2572431, neuroticism (assessed using the NEO-FFI), and coping modes, in the Supplementary Material.

It should be noted that we did not find any evidence of a significant association between rs2572431 and cognitive avoidance. The only trend we found in relation to these variables was that homozygous C-allele carriers showed the highest scores on all cognitive avoidance measures. One possible means of explaining this non-significant finding relates to the pattern of correlations between vigilance and cognitive avoidance with neuroticism. If the overlap between vigilance and neuroticism was greater than the overlap between cognitive avoidance and neuroticism, we might be more likely to expect an effect of rs2572431 on vigilance, instead of an effect on cognitive avoidance. This does not appear to be the case, however, given the magnitude of the correlations reported in the introduction of Egloff and Krohne’s (1998) study (VIG: 0.29 to 0.33 vs. CAV: −0.37 to −0.50). Hence, this explanation does not appear to be able to account for our present findings. It should be noted that the correlation between vigilance and neuroticism is positive, whereas that for cognitive avoidance and neuroticism is negative. rs2572431 might therefore only impact on vigilance due to being positively linked with negative emotionality (as neuroticism), or it is possible that vigilance and neuroticism are sharing part of the variance that does not co-vary with cognitive avoidance.

A further possible explanation for the absence of a significant association between cognitive avoidance and rs2572431 could simply be that the complex interplay between personality, coping, and genetics is not substantively explained by just one genetic variation, but rather by many hundreds of genetic variations that impact on individual differences in coping style – all also shaped by the environment. In line with this, Luciano et al. (2018) showed 116 independent gene variants influencing neuroticism in a large-scale study with over 329,000 participants. In addition to this, Dunn and Conley (2015) described the importance of genetic interactions when focusing on the heritability of coping, hence future studies should therefore take epistasis into account by analyzing different polymorphisms, particularly when dealing with rs2572431 in the context of cognitive avoidance.

On a molecular level, the question arises as to what impact rs2572431 has on the biochemistry underlying individual differences in vigilance. Unfortunately, this is not well understood so far. The rs2572431 polymorphism is located on chromosome 8 (8p23.1) and can be used to track an inversion (*8p23-inv*, Salm et al., 2012) (e.g., Herva and de la Chapelle, 1976; Tian et al., 2008). Okbay et al. (2016) suggested that the molecular effects of the inversion (in linkage disequilibrium with rs2572431) in chromosome 8 on neuroticism could be due to the relocation of sequences that play an important role in regulation of gene expression. The same regulatory changes could possibly play a role in the biological background of vigilance.

Returning to the psychoanalytic approach to defense mechanisms, personality and psychological health, one could further ask how we integrate psychotherapy with this, and therefore also consider the potential modification of an individual’s defense mechanisms with the present molecular genetic findings. Firstly, the environment clearly plays an important role in understanding individual differences in coping modes, as underlined with the results from the twin studies mentioned earlier. An environmental influence, such as the experience of psychotherapy, might result in the modification of an individual’s coping style (e.g., Perry and Bond, 2012). Secondly, recent conceptions of understanding individual differences in personality and related coping modes usually refer to the complex interplay between genetic and environmental factors (Montag and Reuter, 2014). As a consequence, both factors are typically not seen as distinct entities, but rather interacting factors. It would therefore be interesting to understand exactly how the genotypes of rs2572431 interact with different environments to help form human personality. Thirdly, mounting evidence suggests that epigenetic changes can occur due to psychotherapy (e.g., Perroud et al., 2013; Yehuda et al., 2013). Therefore, a response to psychotherapy seems to co-occur with changes of the epigenome, hence changes in gene activity, and most likely with relevant changes on chromosome 8 where our SNP is located. A better understanding of these mechanisms could help to find promising new targets to monitor and understand therapeutic processes, and to evaluate therapeutic outcomes.

A limitation of the current study is the unbalanced proportion of males and females in the sample. It is possible that having a more gender balanced sample would have allowed us to detect gene-by-gender associations, which we were not able to observe in our data. As gender differences in the manifestation of neuroticism, cognitive avoidance and vigilance are well known (e.g., Egloff and Krohne, 1998; Goodwin and Gotlib, 2004), a gene-by-gender interaction would likely be observed with sample sizes greater than in the current study. We may not have been able to detect these effects in the current data because of the relatively small number of males included in the sample.

The homogeneity of the current sample with regard to age and occupational background could also be seen as a limitation of this study. Differences across age in the use of coping strategies have long been debated in the literature (e.g., McCrae, 1982; Felton and Revenson, 1987), and education and profession may also relate to coping. A more heterogeneous sample, allowing for the possibility of examining subsets of participants, e.g., with respect to age, would have allowed a more detailed analysis of these issues. In particular, the issue of the malleability and maturation of defense mechanisms across age is something that should be looked at more closely. It should be noted, though, that age was not significantly associated with coping modes in the current study. We highlight this latter point because although the sample in the current study was relatively young overall, a standard deviation of 7.19 was found in the data.

Finally, we should highlight that the studies of Okbay et al. (2016) and Luciano et al. (2018) were conducted with sample sizes between 170,910 and 329,821. A similar sample size would, of course, have been preferable here, and would have strengthened confidence in our findings. Nevertheless, we are confident in the general approach we pursued in this study; that is, testing a genome-wide based personality SNP in the context of related constructs using smaller samples. This might represent a promising new way forward in Personality Neuroscience, and ultimately this could lead to a new form of the “classic” candidate gene approach. Whereas the “classic” candidate gene approach is heavily based on psychopharmacological studies (Montag and Reuter, 2014; Davis and Montag, 2018; Montag and Davis, 2018), *genome-wide scan based candidate gene* studies could test the most robust SNPs of GWAS in smaller studies to shed more light on these new candidates and their role in psychological phenotypes.

The small effect sizes we detected in this study could be seen as another limitation. As we were only focussing on one genetic variation, however, small effect sizes are not surprising, and can be explained by the notion discussed earlier that vigilance is additionally determined by both environmental factors and potentially hundreds of other genetic factors.

In sum, in the current study we showed that a genetic variation in chromosome 8, the rs2572431 polymorphism, influenced the anxiety-related coping mode of vigilance. This supports the idea of a plausible genetic basis of coping modes/defense mechanisms, and more broadly supports the trait-orientated approach described by Krohne (2001).

Bringing together classic concepts, such as individual differences in anxiety-related coping modes and the modern field of molecular genetics, represents a novel and unique approach to this research area, and we can only hope that other research groups will also join in this interesting endeavor.

## Ethics Statement

This study was carried out in accordance with the local ethics committee at Ulm University, Ulm, Germany with electronic and written informed consent from all subjects.

## Author Contributions

SJ, BL, and CM designed the study. SJ drafted the first version of the manuscript and carried out all the statistical analyses. CM revised the manuscript. CS independently checked the statistical analyses. SJ, BL, CS, and CM collected the samples. All authors approved the final version of the manuscript.

## Conflict of Interest Statement

The authors declare that the research was conducted in the absence of any commercial or financial relationships that could be construed as a potential conflict of interest.

## Footnotes

1 A G to A exchange results in a valin to methionine substitution at codon 158 of the mRNA of the gene coding for the enzyme catechol-*O*-methyltransferase (COMT). This genetic variation is known to impact upon dopaminergic catabolism in the synaptic cleft, especially in the PFC (more information further below in the text).
