# Supplementary material for: rs2572431 Polymorphism on Chromosome 8 Is Associated With Individual Differences in Anxiety Related Coping Modes
Source: Front Psychol. 2019 Jul 9;10:1451. doi: 10.3389/fpsyg.2019.01451 (PMC6629965; doi:10.3389/fpsyg.2019.01451)
Supplement: Supplementary file 1 [file Data_Sheet_1.pdf]

Supplementary Material

1. Neuroticism

Table 1

Correlations between neuroticism (measured via NEO-FFI), cognitive avoidance and vigilance in a subsample of *n* = 638 participants

	Correlation coefficients						
	Neuroticism	CAV-E	CAV-P	CAV-T	VIG-E	VIG-P	VIG-T
Neuroticism	1	-.33**	-.23**	-.34**	.36**	.33**	.39**
CAV-E			.42**	.85**	-.26**	-.13*	-.22**
CAV-P				.83**	-.04	-.36**	-.23**
CAV-T					-.18**	-.29**	-.27**
VIG-E						.57**	.88**
VIG-P							.89**
VIG-T							

Note: * p < .05; ** p <.01, *** p < .001

Neuroticism, gender and rs2572431

Analyses of Variance (ANOVA) were carried out with neuroticism as dependent variable and rs2572431 and gender as independent variables. No correlations between neuroticism and age were detected (r = -.07, p = .06), therefore we didn´t control for age. We did not observe a significant influence of rs2572431 on neuroticism (F(2,632) = 1.86, p = .16, η^2 = .01). But, TT-carrier also showed descriptively the highest neuroticism scores (see Table 2).

Please note two things: Compared to the presented tests in the main body of our paper, power for statistical testing is much lower, because more than 200 participants less filled in the German version of the NEO-FFI by Borkenau & Ostendorf (1993). Beyond this, one could argue to test on a one-sided level in the present context, given i) the findings from the cited GWAS work (Luciano et al., 2018) in the main body of the manuscript and ii) the

findings observed with respect to the moderately correlated construct of vigilance as reported in the main result section of this paper. Such a direct hypothesis would result in a p of .08 (instead of p = .16). In sum, the direction of rs2572431 effect is comparable to what has been observed in the GWAS literature and also in the main body of our work. We believe the non-significant findings in this supplement to be a power issue.

Table 2

Mean scores (standard deviations) for neuroticism by genotype for SNP rs2572431.

	rs2572431 (*n* = 638)		
	CC (*n* = 102)	CT (*n* = 318)	TT (*n* = 218)
Neuroticism	2.67 (.68)	2.78 (.71)	2.79 (.62)

A main effect of gender was found for neuroticism (F(1,632) = 29.91, p<.001, η^2 = .05). Females showed higher neuroticism scores than males.

No gene-by-gender interactions was found (F(2, 632) = 0.24, p = .79, η^2 = .00).

References

Borkenau, P., & Ostendorf, F. (1993). *NEO-Fünf-Faktoren Inventar:(NEO-FFI); nach Costa und McCrae*. Hogrefe.

Luciano, M., Hagenaars, S. P., Davies, G., Hill, W. D., Clarke, T. K., Shirali, M., et al. (2018). Association analysis in over 329,000 individuals identifies 116 independent variants influencing neuroticism. *Nat. Genet. 50*, 6–11.doi: 10.1038/s41588-017-0013-8
